# Parecentesis in Internal Hydrocephalus

**Published:** 1889-06

**Authors:** Samuel Ayres


### PARACENTESIS IN INTERNAL HYDROCEPHALUS.

By SAMUEL AYRES, M. D.

Dr. Ayres reported operation on a boy five years of age, three feet one and a half inches high and weighing forty-three pounds, whose head had been increasing abnormally in size since he was three months old. The chlid, after having frequent convulsions, became imbecile, blind and dumb. The operation improved his condition materially.—ED.

In the performance of the operation great care should be exercised. The chief difficulty lies in our inability to determine which cavity to evacuate. For instance, if the fluid resides in both cavities, and the normal openings between them, through the foramen of Majendie, and those behind the roots of the glosso-pharyngeal nerves be closed by inflammatory exudation, or the presence of a tumor, then to tap only the subdural space would remove the external pressure, and allow such an expansion of the internal fluid as would perhaps lacerate the brain tissue. Or the same effect might be produced by evacuating only the ventricular fluid. This may have been the cause of death in some of the reported cases.

It would seem, therefore, that the safest course to pursue would be that followed in the above, particularly when the cranial sutures are closed—to tap first the ventricles and then the external space.

The operation of tapping for hydrocephalus is not new. It is thought Le Cat first performed it in 1744. West (3d Am. Ed., p. 117) refers to the operation, but states that opinion is divided as to the propriety of the practice. He had up to that time collected fifty-six reported cases from different sources, with four recoveries, but he does not state whether they are of the external or internal form; though the inference is that they were of the former, since compression after operation is referred to.

Watson (p. 391 *et seq.*) gives several cases of the external form, for which tapping was practiced, with variable results, some dying, some recovering, but he speaks of no cures.

Ellis (3d Ed., p. 83) says that puncturing is not applicable to the ventricular form, and he rather discourages its employment in the external. He quotes Churchill, who gives a list of unsuccessful cases. But Ellis admits that the operation has been occasionally successful.

In the Cyclopedia of Practical Medicine (vol. II, p. 500) Folis is quoted as giving the names of twenty-seven writers who had expressed themselves in favor of the operation. Yet he himself with Boerhaave, Dupuytren, Heister, Hecker and Portensclag, regarded it as cruel and useless.

George B. Wood (2d Ed., vol. II, p. 353) says that tapping has been employed by many with uncertain results.

Elliotson (2d Ed., p. 511) states that he never saw a case of the kind, but that if a minute puncture be made and a small quantity of fluid be evacuated at a time, it might be done with safety and with prospects of relief.

Reynolds (System of Med., vol. I, p. 840) quotes Watson, West and Conquest on the subject, the last of whom he states has been the greatest advocate of the operation in this country (England).

Condie (2d Ed., p. 400) refers directly to the tapplng of the ventricles and points out with remarkable accuracy the procedure. At the same time he admits that he had never seen a case.

Trousseau (Clinical Med., vol. I, p. 890)remarks that the brain has been tapped through the sutures and fontanelles for hydrocephalic accumulation, but he does not seem to think its advantages counterbalance its disadvantages.

Henoch (Handbook for Phys. and Student, p. 116, Wm. Wood & Co., 1882) says of chronic internal hydrocephalus, "At least I have achieved no results whatever, either with mercurial inunction, iodide of potassium or applications of tincture of iodine to the head. Nor can I promise any results from compression of the skull with strips of adhesive plaster, or *from puncture through the fontanelles.*"

Gross (5th Ed., vol. II, p. 123) has practiced paracentesis in two cases, both dying within two days after the operation.

Agnew (vol. II, p. 886) speaks of the benefits of the operation as claimed by West and Conquest, but declares such results have never been obtained in this country. He describes only the tapping of external hydrocephalus when the sutures or fontanelles are not closed.

Meigs and Pepper (7th Ed., 1882, p. 557) refer to West's cases and give one of internal hydrocephalus, upon which they operated, death following in less than forty-eight hours. Examination, however, proved that the case was hopeless, the brain being disorganized at the base and the child, less than three years old, the victim of miliary tuberculosis.

J. Lewis Smith (6th Ed., pp. 448 and 449) advises the performance of the operation, and regards it as simple and devoid of danger, but his remarks apply only to the congenital form, and evidently to those with opened sutures and fontanelles. He makes no reference to tapping in acquired hydrocephalus.

Gowers (A Manual of Diseases of the Nervous System, vol. II, 1888, p. 544) considers evacuation by puncture the most direct, but unfortunately the most dangerous, treatment, and advises that but a small quantity be let out each time, and compression of the skull by elastic bandages be kept up. This procedure, he says, is of course most suitable in external effusion, but it has been employed in the ventricular also, occasionally without ill effect, but with absolute success only in rare instances.

An operation in a different kind of case altogether, but with very similar technique, excepting that in this the dura was incised, is referred to by Senn, an imperfect account of which is obtained from the *Melbourne Age*, a non-professional Australian journal.

This operation was done for an echinococcus cyst in the brain on January 27, 1888. The patient, a girl of sixteen, was chloroformed. A button of bone one inch in diameter was removed from the left temple, the dura incised, and a trocar inserted through the substance of the brain, and the cyst successfully penetrated, a large quantity of fluid coming away.

Thus it will be seen that a diversity of opinion is expressed by authors as to the propriety of paracentesis capitis. But in the light of modern antisepsis, and of a more intimate knowledge of the brain anatomically and phyiologically, it would seem that this operation must take its legitimate place among those which were once conceived to be both impracticable and impossible.

I can find reference to no other case similar to the one above reported, in which tapping was practiced for both ventricular and subdural effusion of the primary or acquired character, the sutures and fontanelles being closed, and trephining being required to permit of the entrance of the trocar.—*Cal. Prac.*

